# Nirsevimab Prophylaxis for Reduction of Respiratory Syncytial Virus Complications in Hospitalised Infants: The Multi-Centre Study During the 2023–2024 Season in Andalusia, Spain (NIRSEGRAND)

**DOI:** 10.3390/vaccines13020175

**Published:** 2025-02-12

**Authors:** David Moreno-Pérez, Aleksandra Korobova, Francisco de Borja Croche-Santander, Ana Cordón-Martínez, Olga Díaz-Morales, Leticia Martínez-Campos, Elena Pérez-González, María del Carmen Martínez-Padilla, Juan Luis Santos-Pérez, Jaime Brioso-Galiana, María Isabel Sánchez-Códez, Jorge Del Diego-Salas, Mario Rivera-Izquierdo, Nicola Lorusso

**Affiliations:** 1 Health and Consumption Department, General Directorate of Public Health and Pharmaceutical Management of Andalusia, 41020 Seville, Spain; dmp.malaga@gmail.com; 2 Department of Paediatrics at the Malaga Mother-and-Child Hospital, Hospital Regional Universitario de Málaga, 29011 Málaga, Spain; 3 Multidisciplinary Group for Paediatric Research, Instituto de Investigación Biomédica de Málaga (IBIMA), University of Málaga, 29010 Málaga, Spain; 4 Faculty of Medicine, University of Málaga, 29071 Málaga, Spain; 5 Service of Preventive Medicine and Public Health, Hospital Universitario San Cecilio, 18016 Granada, Spain; aleksandra.korobova.sspa@juntadeandalucia.es; 6 Service of Paediatrics, Hospital Virgen del Rocío, 41013 Seville, Spain; francisco.croche.sspa@juntadeandalucia.es; 7 Service of Paediatrics, Hospital Regional Universitario de Málaga, 29010 Málaga, Spain; anam.cordon.sspa@juntadeandalucia.es; 8 Service of Paediatrics, Hospital Universitario Reina Sofia, 14004 Córdoba, Spain; olga.diaz.sspa@juntadeandalucia.es; 9 Service of Paediatric Infectious Diseases, Hospital Materno Infantil Infanta Leonor, Hospital Universitario Torrecárdenas, 04009 Almería, Spain; lmartinezcampos@gmail.com; 10 Service of Paediatrics, Hospital Virgen Macarena, 41009 Seville, Spain; elena.perez.g.sspa@juntadeandalucia.es; 11 Service of Paediatrics, Hospital Universitario de Jaén, 23007 Jaén, Spain; mcmartinezpadilla@hotmail.com; 12 Service of Paediatrics, Hospital Universitario Virgen de las Nieves, 18014 Granada, Spain; jlsantosperez@gmail.com; 13 Service of Paediatrics, Hospital Universitario Juan Ramón Jiménez, 21005 Huelva, Spain; jaime.brioso.galiana@gmail.com; 14 Service of Paediatrics, Hospital Universitario Puerta del Mar, 11009 Cádiz, Spain; mscodez1990@gmail.com; 15 General Directorate of Public Health and Pharmaceutical Regulation, Ministry of Health and Consumer Affairs of the Regional Government of Andalusia, 41020 Seville, Spain; jorgedeldiego@juntadeandalucia.es; 16 Department of Preventive Medicine and Public Health, University of Granada, 18016 Granada, Spain; 17 Instituto de Investigación Biosanitaria de Granada (ibs.GRANADA), 18012 Granada, Spain; 18 Centros de Investigación Biomédica en Red de Epidemiología y Salud Pública (CIBERESP), 28029 Madrid, Spain; 19 Service of Surveillance and Occupational Health, General Directorate of Public Health and Pharmaceutical Regulation, Ministry of Health and Consumer Affairs of the Regional Government of Andalusia, 41020 Seville, Spain; nicola.lorusso.sspa@juntadeandalucia.es

**Keywords:** respiratory complications, disease severity, tertiary prevention, clinical effectiveness, monoclonal antibodies, immunisation, paediatric

## Abstract

Background: Nirsevimab was indicated in a population level for all infants < 6 months during the 2023–2024 season in Andalusia (southern Spain). Our aim was to analyse the effect of nirsevimab in the reduction in complications in infants hospitalised for RSV bronchiolitis. Methods: A retrospective observational cohort study was conducted in nine relevant hospitals from all provinces of Andalusia, a region with over 9 million inhabitants. The study sample included 222 children, divided into two groups: infants administered with nirsevimab for passive immunisation (exposure) and infants not administered with nirsevimab. Clinical outcomes were analysed, including the use of respiratory support, the need for admission to paediatric intensive care unit (PICU), and duration of hospitalisation. Bivariate analyses were performed, and multivariable logistic regression models were designed to calculate adjusted odds ratios (ORa), and Cox regression models to calculate adjusted hazard ratios (HRa). Results: Bivariate analysis showed an association between passive immunisation with nirsevimab and a lower frequency of numerous outcomes. After adjustment for relevant covariates, multivariable models showed that the exposure (nirsevimab) reduced nasal cannula use by 64% (13–85%), invasive or non-invasive mechanical ventilation by 48% (1–73%), PICU admission by 54% (14–75%), length of hospitalisation by 30% (8–47%), and length of nasal cannula by 31% (7–49%). A higher risk of co-infection was observed in those immunised (aOR = 3.42, 95%CI: 1.52–7.68). Conclusions: Passive immunisation with nirsevimab may decrease the severity of RSV bronchiolitis in infants requiring hospitalisation, thus contributing tertiary prevention that extends beyond the prevention of RSV infection.

## 1. Introduction

Human respiratory syncytial virus (RSV) is the leading cause of bronchiolitis in infants worldwide [1], which can lead to hospitalisation and death [1,2]. The hospitalisation rate due to RSV is notably higher in infants under one year of age compared to other age groups [1,2].

Currently, effective vaccines are available for active immunisation in pregnant women and individuals aged 60 years or older [3,4]. However, no RSV vaccines are currently approved for paediatric use. In 2022, the European Medicines Agency (and, subsequently, the Spanish Agency for Medicines and Health Products) authorised the use of nirsevimab (Beyfortus), a long-acting recombinant human monoclonal antibody, which has been demonstrated to neutralise both RSV-A and RSV-B strains [5]. A single dose of 50 or 100 mg, according to the infant’s weight, can provide protection for at least 5 months [5]. Clinical trials evaluating the efficacy and safety of nirsevimab have shown favourable outcomes, with adverse reactions categorised as rare according to the MedDRA system organ class [5,6,7]. Spain is among the pioneering countries to implement a systematic passive immunisation programme against RSV using nirsevimab in infants [8]. In Andalusia (a region of southern Spain with over 9 million inhabitants), the nirsevimab campaign for the 2023–2024 season started on 1 April 2023, and concluded on 31 March 2024, for all infants under 6 months of age [8]. After the close of the campaign in Andalusia, the coverage for nirsevimab in children born during the season was 93.3% [9].

As one of the first areas in the world to establish systematic passive immunisation with nirsevimab in infants at a population level with a successful coverage, the assessment of the clinical impact of the measure through observational studies seemed highly recommended. To date, several studies have been published on the effectiveness of nirsevimab in primary prevention (reduction in RSV infection) [7,10,11] or reduction of hospitalisation [12,13,14]. Nevertheless, a scarcity of studies has been published on other outcomes related to disease severity (complications in RSV-hospitalised children, such as length of hospitalisation, use of respiratory support devices, or need for intensive care) which also measures the clinical effectiveness of nirsevimab at a tertiary prevention level.

The aim of this study was to evaluate the effectiveness of passive immunisation with nirsevimab and associated factors in reducing the severity of the disease in infants (aged < 6 months) hospitalised with RSV bronchiolitis in Andalusia.

## 2. Materials and Methods

The findings of this study were reported following the strengthening the reporting of observational studies in epidemiology (STROBE) guidelines [15].

### 2.1. Study Setting and Design

The NIRSEGRAND (Nirsevimab-Gravedad-Andalucía; Nirsevimab-Severity-Andalusia in Spanish) study is a retrospective, longitudinal, observational cohort study which included infants born on or after 1 April 2023 who were hospitalised due to RSV bronchiolitis in Andalusia. This study included the hospitals with the highest number of hospital admissions and paediatric intensive care unit (PICU) admissions in each of the provinces of Andalusia. Thus, this multicentre study included all admissions of infants aged < 6 months due to RSV during the 2023–2024 campaign in 9 hospitals: University Hospital Torrecárdenas (Almería); University Hospital Puerta del Mar (Cádiz); University Hospital Reina Sofía (Córdoba); University Hospital Virgen de las Nieves (Granada); University Hospital Juan Ramón Jiménez (Huelva); University Hospital of Jaén (Jaén); University Regional Hospital of Málaga (Málaga); University Hospital Virgen del Rocío (Sevilla); and University Hospital Virgen Macarena (Sevilla). The observation period extended from 1 October 2023 to 29 February 2024. RSV infection was confirmed by a positive PCR test from a respiratory sample collected within 10 days before or up to 3 days after the date of hospital admission. Additionally, bronchiolitis was defined by clinical signs of lower respiratory tract infection, apnoea, sepsis, or a compatible severe acute respiratory infection presentation.

### 2.2. Sources of Information and Variables

The data were collected through a search in the clinical documentation services of the 9 hospitals involved in the study. Data were collected from the electronic medical records systems of the Public Healthcare System of Andalusia.

The exposure was nirsevimab status (previous administration with nirsevimab at least 4 days before hospital admission). Therefore, infants that had received nirsevimab up to 4 days before hospital admission were considered as passively immunised (exposed), and infants that had not received nirsevimab before admission, or that received nirsevimab during the 4 days previous to admission were considered as not immunised (not exposed), given the incubation period of the agent and the time needed to reach protective plasma levels.

The outcomes analysed were measured in terms of the need for respiratory support (of any type), conventional nasal cannula, high-flow oxygen therapy, mechanical ventilation (invasive or non-invasive), invasive mechanical ventilation, nasogastric tube, intravenous access, antibiotic use, chest imaging tests, co-infection (viral PCRs measured at admission, or any bacterial or viral infection identified through PCR or respiratory culture during hospitalisation), admission to PICU, and mechanical ventilation in PICU. In addition, the duration of outcomes was also collected; total length of hospitalisation, length of respiratory support, length of nasal cannula, length of high-flow oxygen therapy, length of mechanical ventilation, and length of PICU hospitalisation.

Other variables collected were sex, hospital of origin, age at admission (in days), use of FilmArray in the diagnosis of RSV infection, and baseline diseases at admission.

Information collected included baseline demographic and clinical data, clinical symptoms, laboratory and radiologic findings, treatment, and patient outcomes, recorded using standardised forms.

### 2.3. Statistical Analysis

First, descriptive analyses were performed to describe the characteristics of the study sample. Categorical variables were presented as frequencies and percentages, and quantitative variables were presented as means and standard deviations or median and interquartile ranges where appropriate. Shapiro–Wilk tests were used to verify the normal distribution of each quantitative variable.

Second, a bivariate analysis was conducted between the exposure variable (passive immunisation with nirsevimab) and each of the outcome variables. To evaluate associations with categorical outcomes, chi-square or Fisher’s exact test was applied depending on data distribution. For quantitative (temporal) outcomes, the Mann–Whitney test was used. Subsequently, cumulative incidence (CI) rates and crude risks ratios (cRR) were calculated. The prevention fraction (PF) was used as a measure of vaccine effectiveness (impact of the exposure), calculated using the formula [(RR − 1) × 100]. The calculation was restricted to outcomes that demonstrated a protective RR association. Nevertheless, CIs present a weakness, as it assumes that the risk of developing the effect is constant throughout the study period (hospitalisation), which is usually not true.

Consequently, incidence rates (IR) were also calculated for each outcome as the number of new cases of each outcome during hospitalisation/total time the population was at risk for this outcome during hospitalisation (sum of total length of hospitalisation for patients that did not develop the outcome plus time from admission to outcome development for patients that developed each outcome). Incidence rate ratios were then calculated comparing the IR of the exposure and non-exposure groups and, subsequently, a PF was calculated from this data [(IRR − 1) × 100].

Third, multivariable analyses were performed for categorical dichotomous outcomes. Before applying logistic regression models, the conditions for application were verified. Crude odds ratios (cOR) and adjusted odds ratios (aOR), along with their 95% confidence intervals (95%CI), were estimated. The dependent variable for each model was each outcome, and the independent variable was the exposure (passive immunisation with nirsevimab). Adjusted models included relevant variables as covariates: sex, age (in days) at admission, hospital, and baselines diseases. Adjusted prevention fractions (aPF) were subsequently calculated, using the formula [(1 − aOR) × 100]. The calculation was restricted to outcomes that demonstrated a protective adjusted odds ratio (aOR) association.

Fourth, survival analyses were conducted to assess temporal outcomes (time to the occurrence of relevant clinical events, e.g., time to discharge which is the total length of hospitalisation). We estimated Kaplan–Meier curves to assess the cumulative probability of event-free survival over time, comparing the exposed and non-exposed groups. Differences between survival curves were evaluated using log-rank tests. Additionally, multivariable Cox regression models were applied using sex, age (in days) at admission, hospital, and baselines diseases for adjustments. The results were expressed as hazard ratios (HR) and their respective 95%CI. Before applying these models, the proportionality of risks, absence of multicollinearity, and appropriate model specifications were verified, ensuring the validity and reliability of the results obtained. All statistical analyses were performed using R version 4.1.2 and Stata (StataCorp^®^ version 15.0, College Station, TX, USA).

### 2.4. Ethical Considerations

All data used were anonymised and potentially identifiable variables were removed from analyses. This study complied with ethical requirements established by the Declaration of Helsinki on human research. The study was approved by the Biomedical Research Ethics Coordinating Committee of Andalusia (CCEIBA) on 28 June 2024, (registration code 202499907099520).

## 3. Results

### 3.1. Characteristics of the Sample

**Figure 1:**
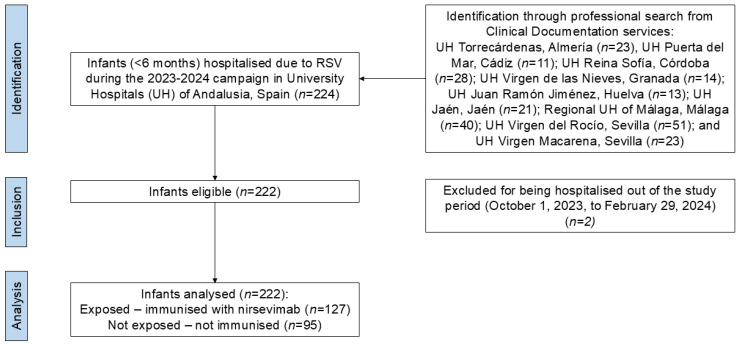
Flow chart of the study sample selection.

The study sample included 222 infants, 127 (57.2%) exposed and 95 (42.8%) not exposed to nirsevimab administration. Figure 1 shows the flow chart of sample selection.

The general characteristics of the sample of infants hospitalised due to RSV are summarised in Table 1. Only one infant was hospitalised within the 4 days after receiving nirsevimab and was considered non-exposed. Of the total, 133 (59.9%) were male, with a higher proportion of males in the immunised group compared to the non-immunised group. The hospital with most admissions was UH Virgen del Rocío, Seville (*n* = 50). More details on hospital characteristics and their reference populations are presented in Supplementary Table S1.

The median age at admission was 66 days (IQR = 55.0) in the exposed group, and 45 days (IQR = 99.5) in the non-exposed group. The use of the FilmArray^®^ diagnostic tool was more frequent in the exposed group (22.1%) than in the non-exposed group (16.8%). Finally, most infants did not present any baseline disease, but 13 (5.9%) infants had underlying conditions, with higher frequency in the exposed group (7.9% vs. 3.2%). The most frequent condition was congenital heart disease (3.2% of the sample).

### 3.2. Bivariate Analysis

Table 2 shows the bivariate associations between exposure (passive immunisation with nirsevimab) and the outcomes analysed during hospitalisation. Briefly, previous nirsevimab administration was associated with less frequent use of a nasal cannula, mechanical ventilation, a nasogastric tube, PICU admission, lower total length of hospitalisation, and a higher frequency of co-infections. The specific co-infections identified (mostly viral) are detailed in Supplementary Table S2.

Table 3 presents the results of cumulative incidences (CI) stratified by exposure group, crude risk ratios (cRR), and the preventive fraction calculated to measure the potential impact of nirsevimab (clinical effectiveness). Nirsevimab showed protection for several outcomes, including the need for respiratory support, cRR = 0.92 (95%CI: 0.84–1.00), PF = 8.4% (95%CI: 0.0–16.1%); nasal cannula, cRR = 0.88 (95%CI: 0.79–0.98), PF = 12.1% (95%CI: 2.0–21.3%); nasogastric tube, cRR = 0.75 (95%CI: 0.56–0.99), PF = 25.2% (0.0–43.6%); and PICU admission, cRR = 0.65 (95%CI: 0.48–0.87), PF = 35.1% (95%CI: 13.1–51.5%).

Table 4 shows the results on incidence rate ratios. For this analysis, the outcomes associated with passive immunisation with nirsevimab were mechanical ventilation, IRR: 0.43 (95%CI: 0.29–0.66), PF = 56.7% (95%CI: 34.1–71.5%), and PICU admission, IRR: 0.49 (95%CI: 0.33–0.73), PF = 50.8% (95%CI: 27.0–66.9%).

### 3.3. Multivariable Analysis of Dichotomous Outcomes

Table 5 presents the results of the multivariate logistic regression models. The exposure (passive immunisation with nirsevimab) was associated with less frequency of need for a nasal cannula, aOR = 0.36 (95%CI: 0.15–0.87), aPF = 64% (95%CI: 13–85%), mechanical ventilation, aOR = 0.52 (95%CI: 0.27–0.99), aPF = 48% (95%CI: 1–73%), and PICU admission, aOR = 0.46 (95%CI: 0.25–0.86), aPF = 54% (95%CI: 14–75%). In addition, passive immunisation with nirsevimab was associated with a higher frequency of co-infections during hospitalisation (aOR = 3.42, 95%CI: 1.52–7.68).

### 3.4. Survival Analysis for Temporal Outcomes

The median time from parental detection of symptoms to hospital admission was 4 days (interquartile range, IQR = 3–5 days) for both groups (exposed and non-exposed to passive immunisation with nirsevimab) (*p*-value = 0.807 of Mann–Whitney test).

**Figure 2:**
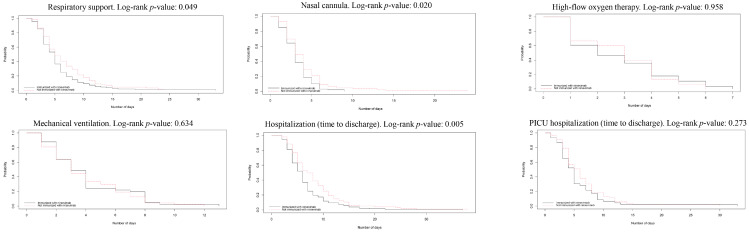
Kaplan–Meier curves for temporal outcomes stratified by exposure to nirsevimab (X axis: number of days; Y axis: probability).

Figure 2 shows the Kaplan–Meier curves and the results of log-rank tests for survival analysis stratified by exposure group. Infants administered with nirsevimab showed less time of respiratory support (*p* = 0.049), a nasal cannula (*p* = 0.020), and hospitalisation time (time to discharge) (*p* = 0.005).

The adjusted Cox regression models (Table 6) showed that nirsevimab was associated with a 30% reduction (95%CI: 8–47%) in total hospitalisation time and a 31% reduction (95%CI: 7–49%) in the length of nasal cannula.

## 4. Discussion

In this cohort study based on a real-life preventive universal intervention with nirsevimab in infants aged < 6 months conducted in Andalusia (southern Spain) during the 2023–2024 season, we showed interesting findings on tertiary prevention. Infants administered with nirsevimab showed less frequent need for a nasal cannula, mechanical ventilation, PICU admission, and a shorter duration of total hospitalisation and nasal cannula use. These data support the idea that nirsevimab is a useful preventive measure beyond prevention of RSV infection or hospitalisation.

The use of nirsevimab for universal prevention of infants started in 2023, and Spain was one of the first countries to establish this strategy and, consequently, to collect real data on the potential impact of the measure. As so, some regions of Spain published their data, like Galicia [16], Valencia, Murcia or Castilla y León [17], Madrid [18], or Catalonia [19] among others. National data pointed to a 74–75% reduction in hospitalisations in infants that received nirsevimab [20]. At a population level, the incidence of RSV during the 2023–2024 campaign in Spain was similar to previous seasons, with lower frequency in infants, with a dramatic decrease in hospitalisation rates for infants aged < 1 year [21], probably due to immunisation strategies in several autonomous communities, including the use of nirsevimab. To date, some recent meta-analyses have showed the effectiveness of nirsevimab in the prevention of RSV infection [10,11] and reduction of hospitalisation rates due to RSV infection [12]. Nevertheless, there is a scarcity of results on outcomes during hospitalisation that could also provide relevant evidence on the potential benefits of nirsevimab in the prevention of disease severity. In this line, Ares-Gómez et al. showed an 86.9% (69.1–94.2) reduction in lower respiratory tract infections requiring oxygen support [16]. Coma et al. showed protection for ICU admission, with an adjusted HR of 0.099 (95%CI: 0.041–0.237) [19]. A study conducted in the region of Madrid showed a 94.4% (95% CI: 87.3 to 97.5) reduction in ICU admission at 30 days [18]. These associations are even stronger than what we showed because both studies [18,19] compared a population-based cohort of infants, whilst we compared a cohort of infants hospitalised due to RSV (therefore, their risk of ICU admission is much higher than in the general population and differences between groups decreases due to sample selection). A pooled analysis of randomised controlled trials also showed benefits in severe disease development or antibiotic prescription, among other outcomes [6]. Recently, a multicentre study conducted in France in infants aged < 1 year showed reduced oxygen supplementation compared to patients not receiving nirsevimab (20.2% vs. 30.6%) and 1-day shorter hospitalisation [22], data similar to those observed in our study.

One of the most surprising findings of this study was the higher frequency of co-infection in infants administered with nirsevimab. This result could be interpreted from several perspectives. Firstly, protection with nirsevimab against RSV infection may increase the risk of other respiratory opportunistic infections but there is no plausible biological mechanism to justify this fact, to the best of our knowledge, according to the current literature. A reason for reduced co-infection in the non-exposed group could be due to difficulty in sampling patients with increased symptoms, but the severity of the symptoms was not collected in this study. Secondly, although some medications reduce the severity of the primary viral disease, they may create a favourable environment for the proliferation of other pathogens, as seen in co-infection studies with viruses such as influenza [23]. Thirdly, the immunological status could influence susceptibility to various infections [24], but this fact would affect both exposed and non-exposed infants. Fourthly, the possibility of parental laxity regarding basic hygiene measures for the prevention of respiratory infections, in the case of receiving nirsevimab. Fifthly, another possible explanation, in our opinion the most likely, is due to the existence of methodological biases. A selection bias due to the inclusion of hospitalised patients cannot be discarded. If nirsevimab reduces the severity of RSV infection, then the need for hospitalisation of an immunised infant is more likely to be due to the existence of other concomitant causes such as infections. It is also possible that infants selected to receive nirsevimab might have characteristics that predispose them to co-infections, including unmeasured confounders. Finally, there could exists a differential surveillance bias if microbiological tests are more frequently requested for immunised infants, possibly due to physicians’ misunderstanding of receiving RSV patients who had been administered with nirsevimab, in search of other pathogens that could explain the clinical presentation. We observed a slight tendency to perform more tests in this group, but not sufficient to completely explain the differences in co-infections. Of note, although infants administered with nirsevimab showed a higher frequency of co-infections, those infections were not associated with any adverse outcomes; instead, this subgroup showed less frequent PICU admission, mechanical ventilation, and a shorter total length of hospitalisation, which suggest that this finding might not be relevant for disease severity. In any case, future studies specifically designed to analyse this association are required.

We assumed that infants were not correctly immunised with nirsevimab unless they had received the passive immunisation at least 4 days before hospital admission. That decision was adopted considering the incubation period of the agent and the unknown time needed to reach adequate plasma levels after administration of nirsevimab. Some authors used 1 day to consider infants as protected [18] and others used up to 7 days [25]. At any rate, only one infant in our cohort was immunised during the 7 days before hospitalisation; thus, in our study the decision of choosing a cut-off point between 1 and 7 days (4 days in our case) would have not changed the results of the study.

We ascertained that none of the mothers of the infants included in the study had received RSV vaccination during pregnancy.

This study has certain limitations. We only included infants hospitalised due to RSV. As previously commented, non-population-based studies intrinsically have a certain degree of selection bias. In addition, the retrospective nature of data collection implies that we did not have information on potentially relevant variables (such us socioeconomic status, or other vaccines received during the campaign); therefore, residual confounding cannot be discarded. For feasibility reasons, we did not include all hospitals of Andalusia, but we selected those with higher number of admissions and PICU, including at least one hospital from each province. As a cohort study (not experimental design), data on the impact of the measure (passive immunisation with nirsevimab) should be considered cautiously. We showed impact measures such as the preventive fraction, but is should not be interpreted as completely causal due to the limitations exposed in this section. Finally, we did not have data on the length of certain outcomes, which prevented us from performing a survival analysis.

Future systematic reviews and meta-analyses are guaranteed to further understand the potential benefits of nirsevimab administration in infants, and specific studies should analyse data on co-infections. Besides. future long-term clinical trials are required to analyse the potential effects of this measure in future campaigns. We hope that the data presented from Andalusia in this study could help future public health decision making.

## 5. Conclusions

Passive immunisation with nirsevimab in infants < 6 months is associated with benefits in outcomes after hospitalisation due to RSV (respiratory support with a nasal cannula, mechanical ventilation, PICU admission, and the total length of hospitalisation), which suggest that this measure is effective beyond prevention of RSV infection or hospitalisation. Future specific studies should analyse the association of nirsevimab with co-infection and other in-hospital outcomes.

## Figures and Tables

**Table 1:** Baseline characteristics of the sample of infants hospitalised due to RSV during the 2023–2024 campaign in Andalusia, Spain.

Characteristics at Admission	Total	Passive Immunisation with Nirsevimab	
		Yes (*n* = 127)	No (*n* = 95)
Sex, *n* (%)			
Men	133 (59.9)	82 (64.6)	51 (53.7)
Women	89 (40.1)	45 (35.4)	44 (46.3)
University Hospital (UH), *n* (%)			
UH Virgen del Rocío, Sevilla	50 (22.5)	22 (17.3)	28 (29.5)
Regional UH Malaga	39 (17.6)	21 (16.5)	18 (19)
UH Reina Sofía, Cordoba	28 (12.6)	19 (15.0)	9 (9.5)
UH Torrecárdenas Almería	23 (10.4)	10 (7.9)	13 (13.7)
UH Virgen Macarena, Sevilla	23 (10.4)	14 (11.0)	9 (9.5)
UH Jaén	21 (9.5)	16 (12.6)	5 (5.3)
UH Virgen de las Nieves, Granada	14 (6.0)	8 (6.3)	6 (6.3)
UH Juan Ramón Jiménez, Huelva	13 (5.9)	8 (6.3)	5 (5.3)
UH Puerta del Mar, Cádiz	11 (5.0)	9 (7.1)	2 (2.1)
Age at admission in days, median (IQR)	59 (71)	66 (55.0)	45 (99.5)
Use of FilmArray, *n* (%)	44 (19.8)	28 (22.1)	16 (16.8)
Any baseline disease, *n* (%)	13 (5.9)	10 (7.9)	3 (3.2)
Congenital heart disease, *n* (%)	7 (3.2)	6 (4.7)	1 (1.1)

IQR, interquartile range; *n* (%), absolute frequency (relative frequency); UH, university hospital.

**Table 2:** Bivariate associations between exposure (passive immunisation with nirsevimab) and the outcomes analysed during hospitalisation.

Outcomes During Hospitalisation (Follow-Up)	Total	Passive Immunisation with Nirsevimab		*p*-Value
		Yes (*n* = 127)	No (*n* = 95)	
Respiratory support, *n* (%)	198 (89.2)	109 (85.8)	89 (93.7)	0.062 ^1^
Conventional nasal cannula, *n* (%)	187 (84.2)	101 (79.5)	86 (90.5)	0.026 ^1^
High-flow oxygen therapy, *n* (%)	43 (19.4)	28 (22.1)	15 (15.8)	0.245 ^1^
Mechanical ventilation (invasive o non-invasive), *n* (%)	89 (40.1)	41 (32.3)	48 (50.5)	0.006 ^1^
Invasive mechanical ventilation, *n* (%)	7 (3.2)	5 (3.9)	2 (2.1)	0.701 ^3^
Nasogastric tube, *n* (%)	102 (46.0)	51 (40.2)	51 (53.7)	0.045 ^1^
Intravenous access, *n* (%)	121 (54.5)	67 (52.8)	54 (56.8)	0.545 ^1^
Antibiotic use, *n* (%)	40 (18.0)	24 (18.9)	16 (16.8)	0.693 ^1^
Chest imaging diagnostics, *n* (%)	97 (43.7)	49 (38.6)	48 (50.5)	0.076 ^1^
Co-infection, *n* (%)	46 (20.7)	35 (27.6)	11 (11.6)	0.004 ^1^
PICU admission, *n* (%)	99 (44.6)	46 (36.2)	53 (55.8)	0.004 ^1^
Mechanical ventilation in PICU, *n* (%)	38 (17.1)	17 (13.4)	21 (22.1)	0.088 ^1^
Length of PICU hospitalisation, median (IQR)	5 (5)	5 (4)	6 (4)	0.167 ^2^
Total length of hospitalisation, median (IQR)	6 (5)	6 (3.5)	7 (5.5)	0.003 ^2^

^1^ Chi-square test, ^2^ Mann–Whitney test, ^3^ Fisher’s exact test. *n* (%), Absolute frequency (relative frequency); IQR, interquartile range.

**Table 3:** Cumulative incidence, crude relative risks, and nirsevimab effectiveness (preventive fraction) for dichotomous outcomes analysed during hospitalisation due to RSV in infants aged < 6 months.

Outcomes During Follow-Up (Hospitalisation)	CIe (Nirsevimab)	CIo (no Nirsevimab)	RR (IC95%)	Effectiveness, PF (IC95%)
Respiratory support	85.8%	93.7%	0.92 (0.84–1.00)	8.4% (0.0–16.1)
Conventional nasal cannula	79.5%	90.5%	0.88 (0.79–0.98)	12.1% (2.0–21.3)
High-flow oxygen therapy	22.0%	15.8%	1.40 (0.79–2.46)	-
Mechanical ventilation (invasive or non-invasive)	32.3%	50.5%	0.63 (0.37–1.10)	-
Invasive mechanical ventilation	3.9%	2.1%	1.87 (0.35–9.85)	-
Nasogastric tube	40.2%	53.7%	0.75 (0.56–0.99)	25.2% (0.0–43.6)
Intravenous access	52.8%	56.8%	0.93 (0.73–1.18)	-
Antibiotic use	18.9%	16.8%	1.12 (0.63–1.99)	-
Chest imaging tests	38.6%	50.5%	0.76 (0.57–1.03)	-
Co-infection	27.6%	11.6%	2.38 (1.28–4.44)	-
PICU admission	36.2%	55.8%	0.65 (0.48–0.87)	35.1% (13.1–51.5)
Invasive o non-invasive mechanical ventilation in PICU	13.4%	22.1%	0.61 (0.34–1.08)	-

CIe, cumulative incidence in the exposed group (passive immunisation with nirsevimab); CIo, cumulative incidence in the non-exposed group (no passive immunisation with nirsevimab); RR, risk ratio (unadjusted); PF, preventive fraction (as a measure of impact of nirsevimab effectiveness).

**Table 4:** Incidence rate, crude incidence rate ratios, and effectiveness for the dichotomous outcomes analysed during hospitalisation due to RSV in infants aged < 6 months.

Outcomes	IR (Total Sample)		IRe (Nirsevimab Administration)		IRo (no Nirsevimab Administration)		IRR (IC95%)	PF (IC95%)
	Cases/ Patients-Day	IR per 100 Patients-Day	Cases/ Patients-Day	IR per 100 Patients-Day	Cases/ Patients-Day	IR per 100 Patients-Day		
Respiratory support	198/347	57.06	109/209	52.15	89/138	64.49	0.81 (0.61–1.07)	-
Conventional nasal cannula	187/826	22.64	101/478	21.13	86/348	24.71	0.86 (0.64–1.14)	-
High-flow oxygen therapy	43/1563	2.75	28/780	3.59	15/783	1.92	1.87 (1.00–3.51)	-
Mechanical ventilation (invasive or non-invasive)	88/1386	6.35	41/926	4.43	47/460	10.22	0.43 (0.29–0.66)	56.7% (34.1–71.5%)
Invasive mechanical ventilation	7/1731	0.40	5/882	0.57	2/849	0.24	2.41 (0.39–25.27)	-
Nasogastric tube	102/948	10.76	51/521	9.79	51/427	11.94	0.82 (0.56–1.21)	-
Antibiotic use	40/1552	2.58	24/765	3.14	16/787	2.03	1.54 (0.82–2.90)	
PICU admission	99/857	11.55	46/547	8.41	53/310	17.10	0.49 (0.33–0.73)	50.8% (27.0–66.9%)

IRe, incidence rate in the exposed group (nirsevimab administration); IRo, incidence rate in the non-exposed group (no nirsevimab administration); IRR, incidence rate ratio (unadjusted); PF, preventive fraction. Incidence rates were calculated as number of new cases of each outcome during hospitalisation/total time the population was at risk for this outcome during hospitalisation (sum of total length of hospitalisation for patients that did not develop the outcome plus time from admission to outcome development for patients that developed each outcome). No data on dates were available for intravenous access, co-infection, chest imaging tests, and mechanical ventilation in PICU in our database; therefore, incidence rates could not be calculated for these outcomes.

**Table 5:** Multivariable analysis of the adjusted association between nirsevimab and the dichotomous outcomes analysed during hospitalisation due to RSV in infants aged < 6 months.

Outcome	cOR (IC95%)	aOR (IC95%)	Effectiveness. aPF
Respiratory support	0.41 (0.16–1.07)	0.38 (0.14–1.03)	-
Conventional nasal cannula	0.41 (0.18–0.91)	0.36 (0.15–0.87)	64% (13–85%)
High-flow oxygen therapy	1.51 (0.75–3.02)	1.32 (0.58–3.00)	-
Mechanical ventilation (invasive or non-invasive)	0.47 (0.27–0.81)	0.52 (0.27–0.99)	48% (1–73%)
Invasive mechanical ventilation	1.91 (0.36–10.04)	3.52 (0.55–22.56)	-
Nasogastric tube	0.58 (0.34–0.99)	0.62 (0.34–1.13)	-
Intravenous access	0.85 (0.88–1.98)	1.07 (0.57–2.00)	-
Antibiotic use	1.15 (0.57–2.31)	1.26 (0.60–2.66)	-
Chest imaging tests	0.62 (0.36–1.05)	0.73 (0.39–1.35)	-
Co-infection	2.91 (1.39–6.08)	3.42 (1.52–7.68)	-
PICU admission	0.45 (0.26–0.77)	0.46 (0.25–0.86)	54% (14–75%)
Mechanical ventilation in PICU	0.54 (0.27–1.10)	0.74 (0.27–2.05)	-

cOR, crude odds ratio; aOR, adjusted odds ratio; aPF, adjusted prevention fraction.

**Table 6:** Adjusted associations between nirsevimab passive immunisation and temporal outcomes during hospitalisation due to RSV in infants aged < 6 months.

Outcome	Patients	Exposed (Nirsevimab), Days		Not Exposed (No Nirsevimab), Days		Log-Rank Test *p*-Value	cHR (IC95%) for Being Administered with Nirsevimab	aHR (IC95%) for Being Administered with Nirsevimab
	*n*	Median (IQR)	Mean (sd)	Median (IQR)	Mean (sd)			
Total length of hospitalisation	222	6 (4–8)	6.62 (4.58)	7 (5–11)	8.47 (5.79)	0.005	0.68 (0.52–0.89)	0.70 (0.53–0.92)
Length of respiratory support	198	5 (3–6)	5.56 (4.26)	5 (4–9)	6.81 (4.87)	0.048	0.76 (0.57–1.01)	0.75 (0.56–1.01)
Length of nasal cannula usage	187	3 (2–4)	3.24 (1.68)	3 (2–5)	4.12 (3.19)	0.020	0.71 (0.53–0.95)	0.69 (0.51–0.93)
Length of high-flow oxygen therapy	43	2 (1–4)	2.75 (1.86)	3 (1–4)	2.87 (1.64)	0.958	1.04 (0.55–1.97)	1.07 (0.51–2.28)
Length of mechanical ventilation ^1^	89	4 (2–7)	5.03 (4.72)	3 (2–6)	4.35 (3.17)	0.471	1.14 (0.75–1.73)	1.12 (0.71–1.77)
Length of PICU hospitalisation	99	5 (3–7)	5.65 (4.99)	6 (4–8)	6.47 (4.64)	0.273	0.81 (0.54–1.20)	0.76 (0.49–1.18)

aHR, adjusted hazard ratio; cHR, crude hazard ratio; IQR, interquartile range, sd, standard deviation. ^1^ Length of total (invasive or non-invasive) mechanical ventilation.

## Data Availability

Data will be available upon reasonable request to the corresponding author.

## Supplementary Materials

The following supporting information can be downloaded at: https://www.mdpi.com/article/10.3390/vaccines13020175/s1, Table S1: Characteristics of the hospitals and population reference included in the study; Table S2: Specific co-infections identified by exposure (nirsevimab, *n* = 127, no nirsevimab, *n* = 95).

## Abbreviations

The following abbreviations are used in this manuscript:

aPF	Adjusted preventive fraction
aOR	Adjusted odds ratio
cRR	Crude risk ratio
CI	Cumulative incidence
95%CI	95% confidence interval
IR	Incidence rate
IRR	Incidence rate ratio
HR	Hazard ratio
OR	Odds ratio
PF	Preventive fraction
PICU	Paediatric intensive care unit
RR	Risk ratio
RSV	Respiratory syncytial virus
