# Supplementary material for: Nirsevimab Prophylaxis for Reduction of Respiratory Syncytial Virus Complications in Hospitalised Infants: The Multi-Centre Study During the 2023–2024 Season in Andalusia, Spain (NIRSEGRAND)
Source: Vaccines (Basel). 2025 Feb 12;13(2):175. doi: 10.3390/vaccines13020175 (PMC11861336; doi:10.3390/vaccines13020175)
Supplement: Supplementary file 1 [file vaccines-13-00175-s001.zip › vaccines-3437587-supplementary.pdf]

Supplementary Table S1. Characteristics of the hospitals and population reference included in the study.

University Hospital (UH), n (%)	*n* of the sample (infants < 6 months admitted due to RSV)	Reference hospital population *	Number of beds	Number of children (aged 0-4) of the province of reference, 2022 **
UH Virgen del Rocío, Sevilla	50 (22.5%)	564,399	1,534	81,919
Regional UH Malaga	39 (17.6%)	336,968	1,049	68,043
UH Reina Sofía, Cordoba	28 (12.6%)	461,078	1,187	29,734
UH Torrecárdenas Almería	23 (10.4%)	338,000	832	36,360
UH Virgen Macarena, Sevilla	23 (10.4%)	481,296	866	81,919
UH Jaén	21 (9.5%)	350,000	805	23,264
UH Virgen de las Nieves, Granada	14 (6.0%)	439,035	844	36,344
UH Juan Ramón Jiménez, Huelva	13 (5.9%)	264,353	620	21,373
UH Puerta del Mar, Cádiz	11 (5.0%)	211,006	652	48,518

* Inhabitants attended primarily (at a first level). Most hospitals have a higher reference population at a second level (for specific techniques or as a reference for certain procedures or services). The most recent data available (between 2019 and 2024 in all cases) was included.

** Data according to the Spanish National Institute of Statistics (https://www.ine.es/jaxi/Datos.htm?path=/t20/e245/p08/l0/&file=03002.px, accessed on 15 January 2025).

Supplementary Table S2. Specific co-infections identified by exposure (nirsevimab, *n* = 127, no nirsevimab, *n* = 95).

Co-infection detected	Total n	Exposed (nirsevimab), *n* (%)	Not exposed (no nirsevimab), *n* (%)
**Virus**			
Rhinovirus / Enterovirus[1]	13	9 (7.1)	4 (4.2)
Rhinovirus	11	9 (7.1)	2 (2.1)
Adenovirus	7	6 (4.7)	1 (1.1)
SARS-CoV-2	4	3 (2.4)	1 (1.1)
Coronavirus OC43	2	2 (1.6)	0 (0.0)
Metapneumovirus	2	2 (1.6)	0 (0.0)
Bordetella pertussis	2	2 (1.6)	0 (0.0)
Influenza A	2	0 (0.0)	2 (2.1)
Parainfluenza 3	1	1 (0.8)	0 (0.0)
Bocavirus	1	1 (0.8)	0 (0.0)
**Bacteria**			
Moraxella catarrhalis	3	2 (1.6)	1 (1.1)
Klebsiella pneumoniae	2	1 (0.8)	1 (1.1)
Escherichia coli	1	1 (0.8)	0 (0.0)
Mycoplasma pneumoniae	1	0 (0.0)	1 (1.1)
Staphylococcus aureus	1	1 (0.8)	0 (0.0)

[1] Rhinovirus and Enterovirus detected in the same test (not possible to differentiate). The total number of co-infections in the table (*n* = 52) is higher than the infants that showed co-infections in the sample (*n* = 46), because some infants presented several co-infections simultaneously. Given the low sample sizes for each agent, no p-values were calculated.
